# Functional cardiac imaging by random access microscopy

**DOI:** 10.3389/fphys.2014.00403

**Published:** 2014-10-20

**Authors:** Claudia Crocini, Raffaele Coppini, Cecilia Ferrantini, Francesco S. Pavone, Leonardo Sacconi

**Affiliations:** ^1^European Laboratory for Non-Linear Spectroscopy (LENS)Florence, Italy; ^2^Division of Pharmacology, Department “NeuroFarBa,” University of FlorenceFlorence, Italy; ^3^Division of Physiology, Department of Experimental and Clinical Medicine, University of FlorenceFlorence, Italy; ^4^Department of Physics and Astronomy, University of FlorenceSesto Fiorentino, Italy; ^5^National Research Council, National Institute of OpticsFlorence, Italy

**Keywords:** microscopy, fluorescence, calcium imaging, voltage-sensitive dye imaging, optical stimulation, channelrhodopsin

## Abstract

Advances in the development of voltage sensitive dyes and Ca^2+^ sensors in combination with innovative microscopy techniques allowed researchers to perform functional measurements with an unprecedented spatial and temporal resolution. At the moment, one of the shortcomings of available technologies is their incapability of imaging multiple fast phenomena while controlling the biological determinants involved. In the near future, ultrafast deflectors can be used to rapidly scan laser beams across the sample, performing optical measurements of action potential and Ca^2+^ release from multiple sites within cardiac cells and tissues. The same scanning modality could also be used to control local Ca^2+^ release and membrane electrical activity by activation of caged compounds and light-gated ion channels. With this approach, local Ca^2+^ or voltage perturbations could be induced, simulating arrhythmogenic events, and their impact on physiological cell activity could be explored. The development of this optical methodology will provide fundamental insights in cardiac disease, boosting new therapeutic strategies, and, more generally, it will represent a new approach for the investigation of the physiology of excitable cells.

## Biological background

The heart is constituted of excitable and contractile cells, called cardiomyocytes. Intracellular Ca^2+^ fluxes mediate the transduction between the electrical activity of cardiomyocyte membrane (sarcolemma) and the mechanical function of the contractile units (sarcomeres). This process is known as excitation-contraction coupling (ECC) (Bers, 2002). ECC abnormalities lead to severe arrhythmias and contractile dysfunction, the two main features common to all cardiac diseases (Hobai and O'rourke, 2001). Although multifaceted, a shared trait of these abnormalities consists in the loss of the spatiotemporal relationship between membrane voltage and Ca^2+^ fluxes (Gomez et al., 1997). Whether and how this occurs has been studied for decades, but it remains a matter of debate due to the lack of tools and techniques able to shed light on ECC microenvironment.

Every heartbeat is triggered by a depolarizing event, the action potential (AP), which is spontaneously generated in the cardiac pacemaker and propagates through the conductive tissue and the working myocardium. In order to achieve a synchronized activation of all cardiomyocytes, the AP spreads from cell to cell throughout the lattice of interconnections called gap-junctions. Gap-junctions are specialized membrane structures, predominantly located at the longitudinal interdigitated ends of cardiomyocytes (Gourdie et al., 1993; Severs, 1994). They consist of transmembrane proteins (connexins), which form hexameric units with a central pore (connexons), whose ion permeability and conductance are regulated by membrane voltage and intracellular ion concentrations (Revel and Karnovsky, 1967). These gap junctions serve as low resistance electrical pathways that allow the heart to work as a syncytium.

The three-dimensional electrical network, constituted of cardiomyocyte layers in the tissue, has a second order of complexity that is a system of deep sarcolemmal invaginations within each single cell. As a network in the network, these sarcolemmal invaginations occur transversely with a periodicity that corresponds to that of sarcomeres (transverse tubules or t-tubules) and they branch in the longitudinal direction (axial tubules) to form a complex system, named transverse axial tubular system (TATS). Indeed, the TATS of cardiac myocytes has been shown to be a network of interconnecting tubules in which the longitudinal elements (10–20% of the total network) bridge 4–5 consecutive Z-lines and, similarly to the transverse components, form junctions with the SR (Soeller and Cannell, 1999; Asghari et al., 2009). TATS ensures a rapid propagation of the electrical signal toward the cell core (Bu et al., 2009), allowing a fast and synchronous Ca^2+^ release from the intracellular Ca^2+^ stores (Sarcoplasmic Reticulum, SR). Indeed, during each AP, depolarization-activated sarcolemmal Ca^2+^ channels open and allow a small amount of extracellular Ca^2+^ to enter the cell, thus generating an inward current (I_CaL_). Ca^2+^ entered via I_CaL_ triggers the opening of Ca^2+^- activated SR Ca^2+^ channels (Ryanodine Receptor, RyR), which are clustered to form Ca^2+^ release units and allow a massive release of Ca^2+^ from the SR. This cascade phenomenon, referred to as Calcium Induced Calcium Release (CICR), preferentially occurs in the TATS membrane, where most of Ca^2+^ channels are located and the spatial coupling with the SR cisternae is tightly established (Ibrahim et al., 2011). This interplay is so tightly regulated that any spatiotemporal disturbance may generate “waves” or “foci” leading to abrupt arrhythmias (O'neill et al., 2004) or, if persisting, to ineffective ECC and severe contractile failure. However, such a belief is often inferred based on indirect or macroscopic observations rather than on direct measurements of the phenomenon at subcellular level. Thus, many questions are still open. First and foremost, how do structural and functional alterations of TATS and SR affect the spatiotemporal properties of ECC? This question is of utmost importance since TATS and SR alterations are the hallmarks of cardiovascular diseases. While membrane voltage depolarization triggers free intracellular Ca^2+^ elevation in physiological settings, the opposite may occur in diseased cardiomyocytes. Spontaneous Ca^2+^ leakages from the SR in the form of Ca^2+^ sparks and widespread Ca^2+^ waves can generate membrane depolarizations, the so called delayed after depolarizations (DADs) (Marks, 2013). DADs are one of the most important mechanisms underlying arrhythmias in cardiac diseases and their occurrence and features are tightly related to the interplay between SR and TATS. DADs are commonly thought to originate from spontaneous diastolic activation of a number of RyRs, determining a local rise of intracellular Ca^2+^ that under certain conditions may propagate to the rest of the cell forming a Ca^2+^ wave. This spontaneously released intracellular Ca^2+^ is removed by the electrogenic Na^+^/Ca^2+^ exchanger (NCX), which is manly located in TATS membrane, leading to a slow diastolic depolarization (i.e., the DAD) which may reach the threshold for a spontaneous premature AP (Pogwizd et al., 2001; Ter Keurs and Boyden, 2007; Venetucci et al., 2007a,b). Whether TATS structural remodeling “*per se*” plays a role in the generation of DADs and cardiac arrhythmogenesis remains largely debated (Orchard et al., 2013). On one hand, loss of TATS may have an anti-arrhythmic effect: by slowing the velocity of Ca^2+^ waves propagation and disrupting the normal proximity of NCX to RyR, loss of TATS may limit the number of DADs. On the other hand, loss of TATS reduces synchronicity of Ca^2+^ release and myofilament activation, thus promoting Ca^2+^-waves and DADs by electromechanical feedback (Ter Keurs and Boyden, 2007).

When cardiac muscle is damaged locally, such as in the border zone of myocardial infarction, Ca^2+^ waves start near the damaged region and propagate in a coordinated fashion into adjacent tissue (Tanaka et al., 2002; Tsujii et al., 2003). Questions about the mechanism of wave propagation in cardiac tissue are the following: do Ca^2+^ waves propagate from cell to cell or does the barrier for Ca^2+^ diffusion imposed by gap junctions stop them? How do propagated Ca^2+^ waves affect membrane voltage at cell surface or in the TATS? Does structurally remodeled TATS promote or prevent Ca^2+^ waves propagation? These questions, essential to understand cardiac pathophysiology, remain unanswered.

Gap junctions play a pivotal role because they ultimately determine how much depolarizing current passes from excited to adjacent resting (non-excited) regions of the network (Kleber, 2011). The size of a given excited region supplying depolarizing current (“current source”) can match or not the amount of depolarizing current necessary to excite the regions ahead (“current sink”). At all levels of complexity in the network (from the whole heart to the cellular TATS), the velocity and safety of excitation spread are dependent on both active and passive properties of the individual elements and on the connectivity of the network. Again, simple questions have never been experimentally addressed: are gap junctions alone responsible for impulse propagation or might electric field mechanisms account for cell-to-cell conduction as well? How do the electrical properties of TATS affect cell-to-cell conduction in the tissue? How large must a Ca^2+^ wave be to impair the electrical conduction?

## The optical revolution

Light has got the potential to address these questions. The combination of light microscopy with functional reporters, caged compounds and optogenetics offers the possibility to control heart activity and monitor functional responses in a non-invasive manner (Arrenberg et al., 2010; Bruegmann et al., 2010). This optical revolution is motivating the development of new optical methods for light stimulation and detection devoted to explore basic features and anomalies in the spatiotemporal relationship between intracellular Ca^2+^ fluxes and AP propagation. This investigation can be made possible thanks to the development of innovative optical methods capable of probing and stimulating cardiac samples in a multiple site modality (York et al., 2012; Botcherby et al., 2013; Howard et al., 2013; Matsumoto et al., 2014). Among those, in this article we focus on recent developments within the area of acousto-optic deflectors. They have been recently used to rapidly scan laser beams across the sample in random access modality, performing optical measurements of membrane potential and intracellular Ca^2+^ across cell (Crocini et al., 2014). In the next future, the same scanning modality could be also used to optically induce local Ca^2+^ releases and spontaneous electrical activities. The outcome of these local perturbations could be investigated in terms of their intra- and inter-cellular propagation and their impact on the regular AP and Ca^2+^ transient. The spatiotemporal relationship between Ca^2+^ and voltage can be dissected at different hierarchical levels, ranging from single isolated cardiomyocytes to intact heart tissue, in well-defined pathological models.

### Spatiotemporal relationship between Ca^2+^ release and voltage at single-cell level

Recently, an ultrafast random access two-photon microscope (Sacconi et al., 2012) in combination with novel fluorinated voltage sensitive dyes with improved photostability (Yan et al., 2012), has been used to measure APs at multiple sites within the T-tubular system of a cardiomyocyte with submillisecond temporal and submicrometer spatial resolution in real time. The noise in such measurements is generally dominated by the shot noise caused by the limited number of photons being detected. For that reason, it is useful to use a dye with high photostability that can produce more photons before it is bleached, and therefore, can produce fluorescence with intrinsically better signal-to-noise ratio.

**Figure 1:**
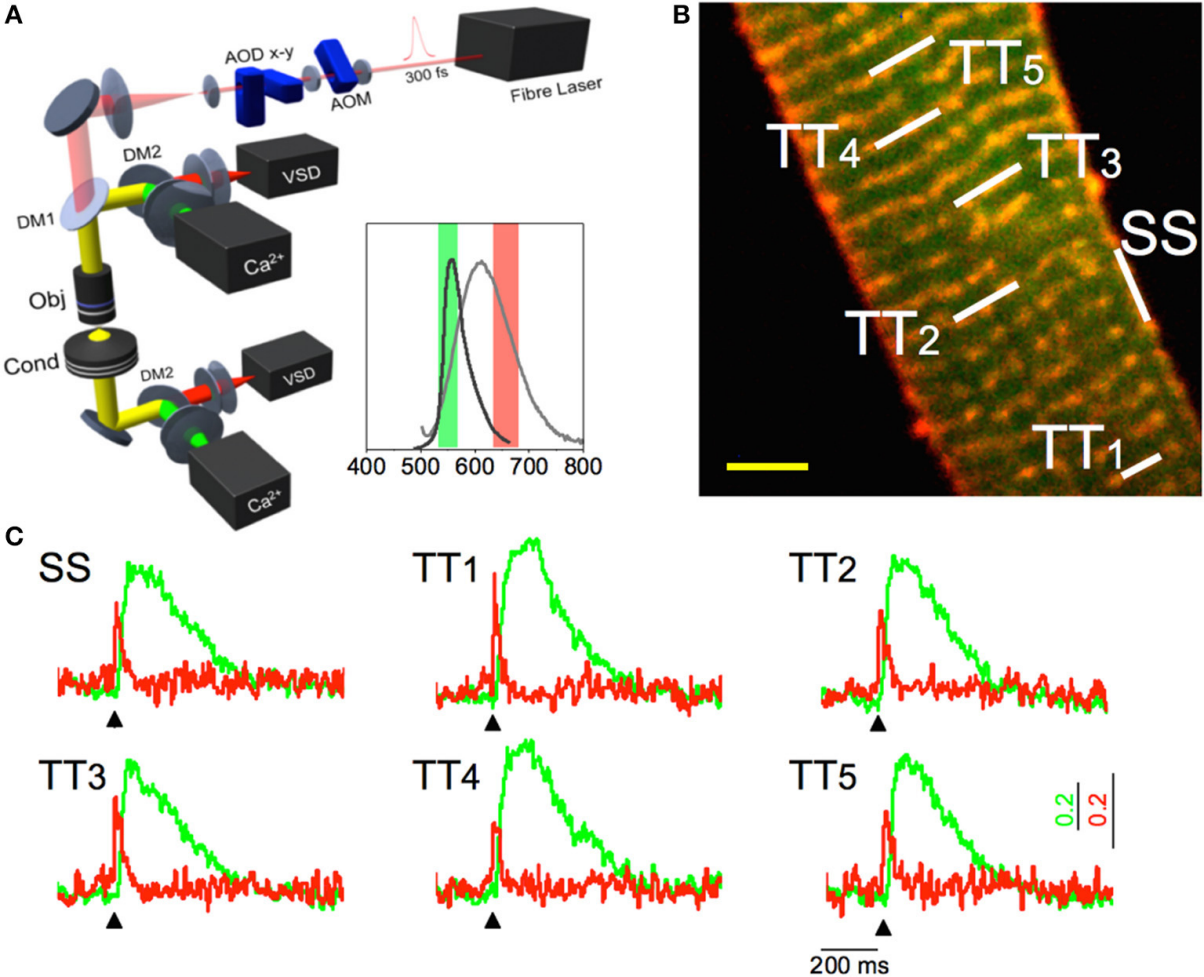
**Multisite voltage and Ca^2+^ recording. (A)** Scheme of the Random Access Multi-Photon (RAMP) microscope. It consists of a 1064 nm fiber laser, an AOM for angular spreading pre-compensation and two orthogonally mounted AODs (AOD-x and AOD-y) for laser scanning. The fluorescence signal is collected in forward and in backward directions using four independent photomultipliers (PMTs), two for the voltage and two for the calcium signals. The inset shows the emission spectra of the Ca^2+^ probe (dark gray) and VSD (light gray) together with the band pass filters used for each channel. **(B)** Two-photon fluorescence (TPF) image of a stained rat ventricular myocyte: sarcolemma in red (di-4-ANE(F)PPTEA) and [Ca^2+^]_i_ in green (GFP-certified Fluoforte). Scale bar: 5 μm **(C)** Real time normalized fluorescence traces (Δ F/F_0_) recorded from the scanned sites indicated in white in panel b: surface sarcolemma (SS) and five T-tubules (TTi). AP is elicited at 200 ms (black arrowheads) by field stimulation. Membrane voltage (red) and [Ca^2+^]_i_ (green). Reproduced and modified with permission from Crocini et al. (2014).

With this approach, it was possible to observe that the tight electrical coupling between different membrane domains is guaranteed only when the tubular system is intact. In fact, the AP propagation into the pathologically remodeled TATS frequently fails and may be followed by local spontaneous electrical activity. These findings provide insights on the relationship between abnormal TATS and asynchronous Ca^2+^ release, a major determinant of cardiac contractile dysfunction and arrhythmias. A simultaneous recording of local Ca^2+^ release and AP can be useful to unravel the consequences of electrical anomalies on the intracellular Ca^2+^ dynamics. To address this challenge, an experimental method capable of optically recording APs in several tubular elements and, simultaneously, the corresponding local Ca^2+^ transients has been recently developed (Crocini et al., 2014). Figure 1 shows an example of an optical recording of voltage and Ca^2+^ release in six different positions across the sarcolemma. In this experiment an isolated rat cardiomyocytes was stained with FluoForte GFP-certified, a Ca^2+^ indicator, and di-4-AN(F)EPPTEA. The random access multi-photon microscope (Figure 1A) was used to simultaneously excite both dyes while band-pass filters were used to select the two distinct spectral ranges of the overlapping fluorescence spectra. The acousto-optic deflectors rapidly scan lines on different membrane segments with a commutation time of 4 μ s between a line and the next. The integration time on each line is about 0.1 ms, leading to a temporal resolution of the order of 0.6 ms for acquiring 6 membrane domains. At the end of one cycle the acousto-optic deflectors return to the initial position and repeats the described cycle, thus probing each site every 0.6 ms. Then, a spectral unmixing procedure was applied to properly uncouple the Ca^2+^ and the voltage signals. As shown in Figure 1, the sensitivity of this optical method is sufficient to detect the presence of an AP and to assess the temporal features of Ca^2+^ transients in real time. The power of this methodology has been demonstrated exploring the role of single TATS elements in determining the correspondent local Ca^2+^ transients as well as a new pro-arrhythmogenic mechanism (Crocini et al., 2014). In fact, the capability of the system in detecting AP and local Ca^2+^ release simultaneously in multiple sites may solve unanswered questions about spatio-temporal relationship between TATS electrical activity and Ca^2+^ release in heart failure.

The next generation of imaging systems can be also implemented with additional ultrafast scanning head for local Ca^2+^ release and/or electrical stimulation. Local Ca^2+^ release can be performed using specific caged compounds (Gurney et al., 1989; Ji et al., 2006), i.e., compounds that release Ca^2+^ at the site of action upon photons absorption and following cleavage of “caged groups.” The photochemical reaction can be very fast, with release of the active species often completed within less than a millisecond. These photochemical reactions can be also induced by two-photon excitation producing highly localized fast Ca^2+^ transients (Rubart, 2004). On the other hand, the electrical stimulation can be achieved by using light-gated ion channels that can be transfected in the plasma membrane of the sample. For example, the photosensitive protein channelrhodopsin-2 (ChR2) has been recently used for photo-stimulating excitable cells (Arrenberg et al., 2010). In fact, under blue light illumination, the cations flux generated through ChR2 produces a rapid membrane depolarization, which can evoke an AP. The two-photon excitation to stimulate ChR2 is more challenging than Ca^2+^ uncaging, mainly due to the low conductance and the long lifetime of the conducting states of ChR2 (Rickgauer and Tank, 2009; Andrasfalvy et al., 2010; Papagiakoumou et al., 2010). In fact, the low conductance and long lifetime make difficult to drive APs by photoactivation with the standard small two-photon excitation volume even by increasing the excitation power. Recent studies suggest that promising non-linear illumination strategies to stimulate ChR2 are based on low excitation density spread over a relatively large excitation area. These requirements could be easily fulfilled using ultra-fast scanning to generate multiplexed excitation patterns that spread the illumination light across an extended area. Furthermore, improved optogenetic tools can now be easily combined with two-photon excitation, allowing light stimulation within single cells (Oron et al., 2012). These new optical implementations will be extremely useful to assess the properties of arrhythmogenic Ca^2+^ waves and to characterize the electrical defects of the tubular system in diseased myocytes. For instance, the rate of spontaneous T-tubular depolarization is rather low (Sacconi et al., 2012), thus a comprehensive assessment of their arrhythmogenic potential is difficult. A direct stimulation of remodeled TATS in cells expressing ChR2 (simulating a spontaneous depolarization) will help understanding whether localized spontaneous T-tubular events play a role in generating or modulating cellular arrhythmias (Ca^2+^ waves and DADs). Moreover, the ability of ultra-fast scanning to stimulate multiple distant TATS regions within the cell will help understanding the amount of single spontaneous events that are required to generate a cell-spanning Ca^2+^ wave.

### Inter-cellular propagations of action potential and Ca^2+^ signal

**Figure 2:**
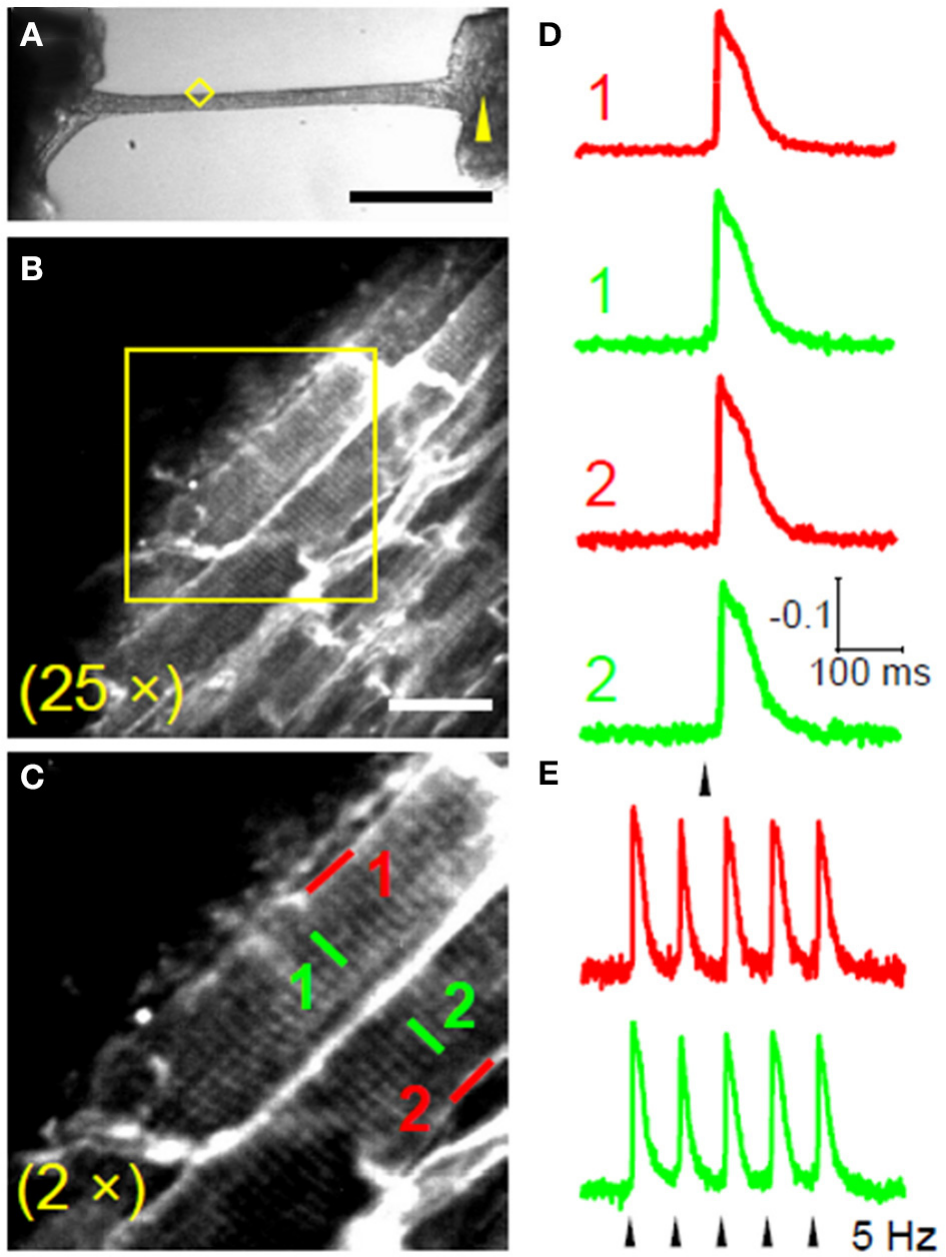
**Intercellular propagation of action potential. (A)** Bright-field image of a rat ventricular trabecula. The yellow arrowhead marks the stimulation site and the yellow diamond encompasses the recording area. (Scale bar: 1 mm.) **(B)** TPF image of the area highlighted in yellow in **(A)**; trabecula stained with di-4-ANE(F)PPTEA (Scale bar: 20 μm.) **(C)** The region in the yellow box of b shows two adjacent myocytes magnified. **(D)** Normalized fluorescence traces from the scanned lines indicated in **(C)**. APs are elicited at 0.2 Hz, corresponding to the black arrowhead. The traces are the average of 10 sequential episodes. **(E)** Normalized fluorescence traces (average of 10 episodes) recorded from SS and TT in cell 1 during stimulation at 5 Hz. Reproduced with permission from Sacconi et al. (2012).

As discussed above, Ca^2+^ waves propagation is caused by local Ca^2+^ release from the SR, followed by diffusion and subsequent activation of adjacent SR Calcium Release units, generating a cascade of events. Ca^2+^ waves are accompanied by DADs (Ter Keurs and Boyden, 2007). Since the average ventricular myocyte is directly coupled to about 11 other myocytes, an afterdepolarization would be immediately suppressed by the large “current sink” unless a sufficient number of neighboring cardiomyocytes also synchronously develop an afterdepolarization at the same beat. Propagation of Ca^2+^ waves from cell-to-cell has been shown to occur at a constant velocity (V_prop_) that ranged from 0.1 to 15 mm/s, a speed that is too slow to be determined by active (1 m/s) or electrotonic membrane conduction (0.1 m/s) (Ter Keurs and Boyden, 2007) but is compatible with a facilitated Ca^2+^ diffusion. Mechanisms underlying Ca^2+^ waves propagation are still poorly understood. However, V_prop_ can increase under certain circumstances, such as increased RyR open probability or in presence of SR Ca^2+^ overload. The faster the V_prop_ the higher the probability that a large area would “nearly simultaneously” be invaded by a propagated Ca^2+^ wave, thus increasing the likelihood of “synchronized” DADs in adjacent cells. Exploiting the advantage of multi-photon excitation, multisite optical recordings can also be performed in multicellular preparation such as intact trabeculae and muscle bundles in which cell-to-cell conduction occurs through sarcolemmal gap junctions (Sacconi et al., 2012; Ferrantini et al., 2014). Figure 2 shows examples of APs recorded in SS and TATS of two adjacent cells. Trabeculae were locally stimulated in a region 2 mm apart from the recording area (Figure 2A), and an 15 ms delay was found between the stimulus and the rapid AP upstroke phase (Figure 2D), in agreement with the expected myocardium conduction velocity at room temperature.

Following a similar approach described above the same excitation wavelength can be used to simultaneously detect voltage and Ca^2+^ in different cells, opening new perspectives for the investigation of inter-cellular propagation of Ca^2+^ waves. On the other hand, the probability to observe a spontaneous Ca^2+^ wave within the limited field of view of laser scanning microscopy is extremely low. Thus, the implementation of a multi-photon Ca^2+^ uncaging system based on random access scanner can be used to induce local elevation of intracellular Ca^2+^ in different position within the scanned region. For example this approach can be exploited to explore how Ca^2+^ perturbation in a single myocyte affects Ca^2+^ propagation to the neighboring cells and how RyR open probability and cytosolic Ca^2+^ buffering affect V_prop_ and DADs “synchronicity” between neighboring cells. We could assess whether premature local elevation of Ca^2+^ in a single cell is able to trigger Ca^2+^ release in the neighboring cells, thus synchronizing Ca^2+^ release to generate a propagated Ca^2+^ wave. Moreover, by inducing a Ca^2+^ wave during regular pacing, we could study how unregulated Ca^2+^ release affects electrical propagation and whether this facilitates the formation of a re-entry circuit or other forms of sustained arrhythmias. Pharmacological interventions such as low dose of caffeine (to increase RyR open probability) or cyclopiazonic acid (to decrease Ca^2+^ buffering) can be administrated to enhance V_prop_. Conversely, pharmacological blockers of cardiac RyR, such as JTV519 (Elliott et al., 2011), could be employed to test the consequeces of RyR desensistization on spontaneous wave propagation. Finally, multicellular preparation dissected from mice carrying mutations of the cardiac RyR with intrinsically higher or lower open probability (Liu et al., 2006; Jones et al., 2008) could be also employed to elucidate how changes of RyR open probability affect local Ca^2+^ dynamics.

## Conclusions

Light microscopy together with functional fluorescent indicators is speeding up the comprehension of biological mechanisms in excitable cells. Cardiac research has been profoundly affected by the development of optical methodologies that allow non-invasive and fast imaging. Ultrafast scanning modalities have made possible multisite membrane potential recordings across single cardiomyocytes and multicellular preparations. More recently, multisite recording of membrane potential and Ca^2+^ has been reported, disclosing in details their spatio-temporal relationship. The scenario is now getting even more exciting. Indeed, light can be employed even to control the two main determinants of cardiac function by using photosensitive ion channels and light-dependent Ca^2+^ caged compounds.

The opportunities that light may provide on cardiac research are not yet fully exploited. New light-based technologies can disclose cardiac function at different levels, from single molecule to the whole heart, as well as provide possible implications for future therapeutic applications.

### Conflict of interest statement

The authors declare that the research was conducted in the absence of any commercial or financial relationships that could be construed as a potential conflict of interest.
